# The HAC Trial (Harmonic for Acute Cholecystitis) Study. Randomized, double-blind, controlled trial of Harmonic(H) versus Monopolar Diathermy (M) for laparoscopic cholecystectomy (LC) for acute cholecystitis (AC) in adults

**DOI:** 10.1186/1745-6215-10-34

**Published:** 2009-05-26

**Authors:** Fausto Catena, Luca Ansaloni, Salomone Di Saverio, Filippo Gazzotti, Federico Coccolini, Antonio Daniele Pinna

**Affiliations:** 1 Emergency Surgery Unit, Department of General and Transplant surgery (Prof A. D. Pinna), S. Orsola Malpighi University Hospital, Bologna, Italy

## Abstract

**Background:**

In the developmental stage of laparoscopic cholecystectomy (LC) it was considered 'unsafe' or 'technically difficult' to perform laparoscopic cholecystectomy for acute cholecystitis (AC). With increasing experience in laparoscopic surgery, a number of centers have reported on the use of laparoscopic cholecystectomy for acute cholecystitis, suggesting that it is technically feasible but at the expense of a high conversion rate, which can be up to 35 per cent and common bile duct lesions.

The HARMONIC SCALPEL(R) (H) is the leading ultrasonic cutting and coagulating surgical device, offering surgeons important benefits including: minimal lateral thermal tissue damage, minimal charring and desiccation.

Harmonic Scalpel technology reduces the need for ligatures with simultaneous cutting and coagulation: moreover there is not electricity to or through the patient Harmonic Scalpel has a greater precision near vital structures and it produces minimal smoke with improved visibility in the surgical field.

In retrospective series LC performed with H was demonstrated feasible and effective with minimal operating time and blood loss: it was reported also a low conversion rate (3.9%).

However there are not prospective randomized controlled trials showing the advantages of H compared to MD (the commonly used electrical scalpel) in LC.

**Methods/Design:**

Aim of this RCT is to demonstrate that H can decrease the conversion rate compared to MD in LC for AC, without a significant increase of morbidity.

The patients will be allocated in two groups: in the first group the patient will be submitted to early LC within 72 hours after the diagnosis with H while in the second group will be submitted to early LC within 72 hours with MD.

**Trial Registration:**

ClinicalTrials.gov Identifier: NCT00746850

## Background

In the developmental stage of laparoscopic cholecystectomy it was considered 'unsafe' or 'technically difficult' to perform laparoscopic cholecystectomy for acute cholecystitis [1,2].

With increasing experience in laparoscopic surgery, a number of centers have reported on the use of laparoscopic cholecystectomy for acute cholecystitis, suggesting that it is technically feasible but at the expense of a high conversion rate, which can be up to 35 per cent [3,4] and common bile duct lesions [5].

The HARMONIC SCALPEL^® ^(H) is the leading ultrasonic cutting and coagulating surgical device, offering surgeons important benefits including: minimal lateral thermal tissue damage, minimal charring and desiccation.

H technology reduces the need for ligatures with simultaneous cutting and coagulation: moreover there is not electricity to or through the patient

H has a greater precision near vital structures and it produces minimal smoke with improved visibility in the surgical field. [6]

In retrospective series LC performed with H was demonstrated feasible and effective with minimal operating time and blood loss: it was reported also a low conversion rate (3.9%). [6]

However there are not prospective randomized controlled trials showing the advantages of H compared to Monopolar Diathermy (MD – the most commonly used electrical scalpel) in LC.

Aim of this RCT is to demonstrate that H can decrease conversion rate compared to MD in LC for AC, without a significant increase of morbidity.

## Methods

The study project is a prospective, randomized investigation. The study will be performed in the Department of Emergency Surgery St Orsola-Malpighi University Hospital (Bologna, Italy), a large teaching institutions, with the participation of all surgeons who accept to be involved in.

The Ethic Committee of St. Orsola Malpighi University Hospital has approved the study protocol on June 24^th ^2008. The study and its Informed Consent form have been judged by the Committee to be ethically and scientifically satisfactory as well as correct and adequate to the aims.

The patients will be allocated in two groups: in the first group the patient will be submitted to early LC within 72 hours after the diagnosis with H while in the second group will be submitted to early LC within 72 hours with MD.

The randomization will be obtained through computer-generated schedule. Blocked randomization is used to ensure close balance of the numbers in each group at any time during the study. The result of this randomization will be sealed in numbered envelopes, inserting inside a cardboard to ensure that they are opaque. After cholecystitis diagnosis if the patient fulfils the inclusion criteria the responsible surgeon will ask the patient to partecipate to the study. If the patient agree, he/she will sign the informed consent. After patient's consent the randomization will be carried out. The responsible surgeon will record the patient name (and number). [7-9]

All eligible patients will be recorded. [10]

### Statistical Methods and Power Calculations

By using StatCal of Epi INFO 2000 software package (Centers for Disease Control and Prevention, Atlanta, GA, USA), a sample size of 21 patients for each group (42 patients for the whole study) has been calculated to reach a confidence level of 95% with a power of 80%, supposing that the hospital stay for LC with H the conversion rate can be reduced from 35% to 3%.

Analysis of data is by intention-to-treat. It will be specified when and how any deviations from randomised allocation, false inclusions, or missing outcomes have been handled. Repeated measures of the outcome will be performed with complete case analyses, potentially excluding individuals with some follow-up data, Eventual missing outcomes will be imputed using baseline values or by assuming that all missing participants have the same risk as the observed participants in the control group.

Data are expressed as numbers (%) and means (SD). The results of the two groups in comparison are analyzed using the Pearson's chi-square test and Fisher exact test, as appropriate, for proportions in case of discrete data. Fisher exact test is used when the data are very unequally distributed among the cells of the table or the expected frequency of any cell is less than 5 or the total N is less than 50. For means in case of continuous numerical data, the independent samples T test and the Mann-Whitney U-test are used, respectively for data normally and non-normally distributed. The data are previously tested for normality by the Kolmogorov-Smirnov test. For multivariate analysis the stepwise logistic regression is applied. A *p*-value of < 0.005 is in any case considered to be statistically significant.

Forward selection has been used as variable selection method; it will include all the baseline variables and all baseline variables are assessed for imbalance. The final model only contain variables with a p-value less than 0.05. Imbalances of baseline variables are defined comparing the groups using t tests for continuous variables and χ^2 ^tests for categorical variables. The adjustments are based upon the stratified logrank test or analysis of covariance. [11-13]The primary outcome is the conversion rate. Secondary outcomes are: intra-operative and post-operative morbidity and mortality, operative time and intra-operative blood losses.

All the secondary outcomes, including morbidity and mortality, operative time and blood losses, will be controlled as well for imbalances, (i.e. age, previous comorbidities, anticoagulant therapies, previous abdominal surgery).

Inclusion criteria are:

• Adult patients (>18 years)

• Clinical (pain, fever > 37.5°C, WBC > 10.000/microL), and ultrasound evidence of cholecystitis

• ASA I-III patients

• Informed consent

• Less than 72 h from the onset

Exclusion criteria are:

• Informed consent refusal

• Choledocholithiasis

• Generalized peritonitis

• Previous abdominal surgical procedures

• Patients with an intra-operative findings of different pathology will be excluded from the study

• Apache II score > 10

### Intervention

Preoperative data collected will include patient demographics and comorbidity conditions (genitourinary, cardiac, pulmonary, gastrointestinal, renal, or rheumatologic) and a detailed history of symptom onset.

The procedure was performed by a surgeon that had performed at least 50 LCs.

On admission, the patients were started on cefotaxime, 2 g IV every 12 h, which was continued postoperatively according to NNISS score.

The standard four-trocar operative technique is used for LC for acute cholecystitis.

When the gallbladder is distended it will be first aspirated. To allow a good hold on the gallbladder larger graspers will be inserted through a 5 mm right lower port. The cystic artery and duct are clip-ligated in the MD group whereas in the H group cystic artery and duct are closed by H. In the H group the surgeon will use only H, whereas in the MD group the surgeon will use only MD. The gallbladder and intraperitoneal "dropped" stones are collected in an endoscopic bag and extracted through the umbilical cannula site, which can be extended. A closed system suction drain is left. Fascial closure is attempted only at the umbilical cannula site. The skin at all the cannula sites are closed with staples. Conversion to laparotomy will be decided by the operating surgeon and each conversion will be motivated.

### Data Collection

Patients' data sheets are generated containing demographic data and preoperative, operative, and postoperative information.

Pre-operative notes concern the history of gallbladder stones, the presence of associated diseases (cardiac, hypertension, diabetes, malignancy), duration of gallbladder complaints (as an indication for the onset of the disease), finding of a palpable gallbladder, temperature, and laboratory results of WBC count, serum bilirubin, gamma GT, CRP, IL-6 and alkaline phosphatase.

Ultrasound findings are also reported.

Operative data of concern are macroscopic findings (of acute cholecystitis, gangrenous cholecystitis, hydrops, and empyema of the gallbladder), the presence of small stones (< 1 cm diameter) or large bile stones (> 1 cm diameter), information regarding perforation of the gallbladder and intraperitoneally "lost" stones, reasons for conversion, and duration of surgery. Postoperative notes of interest included the use of nasogastric tubes and drains, the amount of analgesics used, (evaluation of pain with VAS score), complications, and length of hospital stay.

Complications are classified as surgical infections (wound infection, subphrenic or subhepatic abscess); noninfectious surgical problems (e.g., bile duct injury, hemorrhage); remote infections (urinary or respiratory); and miscellaneous problems (e.g., atelectasis, deep vein thrombosis, AMI, CVA, etc). The collected information are entered into a database as either continuous or categorical variables for statistical analysis. Following the operative procedure, a normal sterile dressing will be applied to cover the abdomen.

A second surgical team, aware of the operative findings but not the surgical dissection instrument, then will assume the care of the patient. Postoperative care and ability to be discharged from the hospital will be determined by the second surgical team. The primary operative team will be in every moment available for emergent consultation.

Patient discharge will be base on good medical practice criteria: 1) apyrexia 2) absence of diseases requiring hospitalisation 3) return of bowel function 4) patient's compliance.

No Placebo drugs are used for this study.

Every patient will be asked to sign the Informed Consent.

In the informed consent form, patients will receive all the information about the study protocol, the confidential nature of personal data and will fill up a questionnaire before signing or refuse.

There will be not inconveniences caused to the patients. No incentives are planned for the patients regarding the operation or the follow-up.

All the medical informations obtained from the patients will be kept confidentially among the research scientists conducting the study.

The patients will be free to withdraw from the study, whenever they want without any obligation.

The study will be stopped in case of newly discovered statistically significant advantages in one group.

The aim of the study is to demonstrate that H can reduce conversion rate compared to MD in LC for AC but also differences in terms of morbidity, mortality, operation time, hospital stay, postoperative pain, return to normal activity will be evaluated.

The primary endpoints of our study will be:

**A) **To evaluate the conversion rate

**B) **To evaluate morbidity, mortality, operation time

**C) **To evaluate hospital stay, postoperative pain, return to normal activity

The onset of any other complications will be recorded intraoperatively, postoperatively, at discharge, at 7-days, 1-month and 6-months.

The side effects that could have observed are not substantially different between the arms of the study.

All the above mentioned data will be recorded in the Case Report Form and later stored in computer database. At the end of the study the final statistical examination will be carried out.

An interim statistical examination of the data will be done every 3 months during the period of patients' inclusion in the study. Then at the end of every completed follow-up period (1-month, 6-months).

The statistical analysis will be carried out using Epi Info 2000, Version 1.1 software package (Dean AG, Arner TG, Sangam S, Sunki GG, Friedman R, Lantinga M, Zubieta JC, Sullivan KM, Smith DC. Epi Info 2000, a database and statistics program for public health professionals for use on Windows 95, 98, NT, and 2000 computers; Centers for Disease Control and Prevention, Atlanta, Georgia, USA, 2000)

No incentives are planned for the patients regarding the operation or the follow-up.

The study will take approximately 6 months – 1 year for the inclusion period. According to the number of AC managed monthly, the duration of the inclusion period can be approximately of 1 year to reach the number of about 42 enrolled patients.

An interim report is planned at the end of any completed follow-up period.

The trial has been registered with the ClinicalTrials.gov Identifier: NCT00746850 .

## Discussion

AC is a common disease. Any improvement in this field will benefit many patients reducing morbidity, mortality, conversion rate, operation time, hospital stay, postoperative pain, return to normal activity and aesthetic result. All our patients will be informed about the study and an informed consent will be obtained. There will not be inconveniences caused to the patients. All the medical informations obtained from the patients will be kept confidentially among the research scientists conducting the study. The patients will be free to withdrawn from the study, whenever they want without any obligation.

## Abbreviations

LC: laparoscopic cholecystectomy; AC: acute cholecystitis; H: Harmonic Scalpel; MD: Monopolar Diathermy; RCT: Randomized Controlled Trial; NNISS: National Nosocomial Infection Surveillance Study; WBC: White blood cells; CRP: C reactive Protein; AMI: Acute Myocardial Infarction; CVA: cerebrovascular accidents.

## Competing interests

Each author has participated sufficiently in the work to take public responsibility for appropriate portions of the content. All authors read and approved the final manuscript and declare no competing interests.

## Authors' contributions

FC, LA have made substantial contributions to conception and design, or acquisition of data, or analysis and interpretation of data; SDS, FG, FC have been involved in drafting the manuscript or revising it critically for important intellectual content. ADP conceived of the study, and participated in its design and coordination and helped to draft the manuscript.
